# Editorial: Intercellular communication and crosstalk in cardiac development and disease

**DOI:** 10.3389/fcvm.2022.1053539

**Published:** 2022-10-07

**Authors:** David T. Paik, Hyeonyu Kim, Edward Lau

**Affiliations:** ^1^Stanford Cardiovascular Institute, Stanford University, Stanford, CA, United States; ^2^Division of Cardiovascular Medicine, Department of Medicine, Stanford University School of Medicine, Stanford, CA, United States; ^3^Division of Cardiology, Department of Medicine, University of Colorado School of Medicine, Aurora, CO, United States

**Keywords:** multi-omics, artificial intelligence, cellular crosstalk, cellular communication, single-cell sequencing

Different cell types within a tissue communicate with each other to orchestrate physiological functions. The mammalian heart contains various cell types including cardiomyocytes, endothelial cells, fibroblasts, smooth muscle cells, and resident immune cells. Whereas, many progresses have been made from the study of bulk tissues (without distinguishing individual cell types) or from isolated cells alone, it has also become clear that many biomedical questions can only be answered by considering the interactions and crosstalk across different cell types. The advent of large-scale omics and single-cell studies now allows increasing power to investigate biological processes in individual cell resolution to discern the contribution of individual cells or cell types to whole-organ physiology. In parallel, systems biology and computational approaches are being developed to harness large-scale data to model intercellular communication across individual cells. These developments hold the promise of transforming our understanding of tissue physiology, homeostasis, and disease pathological mechanisms involving multiple cell types. Accordingly, this Research Topic highlights several articles that reveal some emerging frontiers in this area.

A timely review article by Vera et al. discusses the promise of stem cells, multi-omics big data, and artificial intelligence (AI) of tackling Duchenne Muscular Dystrophy (DMD), the most prevalent type of muscular dystrophy diseases. DMD is caused by mutations that affect the dystrophin protein, which normally anchors the cytoskeleton to the extracellular matrix (ECM) in muscle cells. DMD affects not only skeletal muscle cells, but also cardiac muscle. As the striated muscle cells compensate for the disruption of dystrophin, stressed cells begin to secrete inflammatory cytokines that lead to fibrosis, inflammation, and cell death, suggesting therapeutic strategies may target secondary pathologies that involve cell to cell communications. A single-nuclei sequencing atlas of skeletal muscle in a dystrophic mouse model confirms that crosstalk between myocytes, endothelial cells, and macrophages is potentially responsible for pathogenesis. The article concludes by reviewing the combination of induced pluripotent stem cell (iPSC) models, AI-driven drug screen, and multi-omics approach that is propelling the search of DMD therapeutics.

A review by Qu et al. provides an updated summary of the effects of interactions between endocardial cells and cardiomyocytes on their differentiation and trabeculation during early cardiac development. The authors presented current hypotheses and controversial areas for the origins of endocardial cells and cardiomyocytes with their multipotent precardiac progenitors. In addition, they described the crosstalk between endocardial cells and cardiomyocytes through bone morphogenetic protein (BMP) and Hedgehog signaling during their differentiation. They also introduced their communication during trabeculation through Notch, PlexinD1, Tie2, vascular endothelial growth factor (VEGF), and ECM signaling. This review will provide important insights for understanding congenital heart disease caused by inappropriate endocardial-myocardial interactions by introducing the related pathways and their importance during early cardiac development, particularly during trabeculation.

Intercellular communication is also heavily studied in endogenous tissue repair processes. In the adult heart, following an acute ischemic injury event such as a myocardial infarction, the paracrine role of various non-cardiomyocyte cell types has shown to be critical in triggering dynamic cellular and molecular processes in response to injury. The review by Huang and Huang thoroughly summarizes the origin, recruitment, and characteristics of endothelial progenitor cells and their autocrine and paracrine role in cardiac remodeling and tissue repair.

Hypertension is a major risk factor that leads to coronary artery disease and to kidney disease and failure. A primary research article by Luo et al. included in this Research Topic investigated the effects of aerobic exercise training on renal function in spontaneously hypertensive rats. The group identified that levels of Renalase, a flavin/adenine/dinucleotide-dependent amine oxidase, increased in the blood and skeletal muscle with frequent and moderate aerobic exercise, which was shown to have beneficial effects in attenuating Angiotensin II-induced oxidative stress and apoptosis in human renal cells. On the other hand, Renalase expression was found to be reduced in renal biopsy specimens from hypertensive patients. This study consequently suggests a potential therapeutic role of Renalase in combatting renal damage from hypertension, and that aerobic exercise ameliorates hypertension complications *via* enhanced cell to cell signaling.

Taken together, this Research Topic explores the effects of crosstalk among multiple cell types in cardiac development and regeneration as well as in musculoskeletal and renal diseases. As recent technological advances, including the advent of AI, next-generation sequencing, and single-cell analyses, provide more opportunities to elucidate complex heterotypic cell-cell interactions, further research and attention in this Research Topic will enable comprehensive understanding of their communication. This will greatly benefit the development of novel and promising therapeutic interventions for multifaceted diseases that pose major global public health risks.

## Author contributions

All authors listed have made a substantial, direct, and intellectual contribution to the work and approved it for publication.

## Funding

HK was supported by postdoctoral fellowship from the American Heart Association. EL was supported by NIH awards R00-HL144829 and R03-OD032666. DP was supported by NIH award HL150216 and American Heart Association award 20CDA35260261.

## Conflict of interest

The authors declare that the research was conducted in the absence of any commercial or financial relationships that could be construed as a potential conflict of interest.

## Publisher's note

All claims expressed in this article are solely those of the authors and do not necessarily represent those of their affiliated organizations, or those of the publisher, the editors and the reviewers. Any product that may be evaluated in this article, or claim that may be made by its manufacturer, is not guaranteed or endorsed by the publisher.

